# Predictors of Remission in Severe Childhood Immune Thrombocytopenia

**DOI:** 10.3390/diagnostics13030341

**Published:** 2023-01-17

**Authors:** Chao-Neng Cheng, Yuan-Ning Yang, Yun-Hsuan Yeh, Li-Wen Chen, Jiann-Shiuh Chen, Yung-Chieh Lin

**Affiliations:** 1 Department of Pediatrics, National Cheng Kung University Hospital, College of Medicine, National Cheng-Kung University, Tainan 704302, Taiwan; 2 Department of Pediatrics, College of Medicine, National Cheng-Kung University, Tainan 701401, Taiwan

**Keywords:** acute, children, chronic, immune thrombocytopenia, ITP, predictor, remission

## Abstract

Childhood immune thrombocytopenia (ITP; platelet count < 100 × 10^9^/L) is the most common bleeding disorder in children. A total of 3–5% of children with ITP face a greater risk of bleeding, resulting in significant morbidity and mortality. Childhood ITP is often benign and self-limited; however, children with severe ITP (platelet count < 30 × 10^9^/L) require investigation and monitoring. In addition, 20% of ITP patients may not go into remission (platelet counts < 100 × 10^9^/L by 12 months after diagnosis) and may develop chronic ITP. The early identifying predictors associated with the resolution of severe ITP at the time of diagnosis may be helpful for family guidance. However, there is still controversy about the associations between the clinical factors at the time of initial diagnosis and the definitions of disease remission assessed at different timepoints after diagnosis. This retrospective study aimed to analyze the shared clinical factors among the disease remission definitions at three arbitrarily set timepoints—3, 6, and 12 months after diagnosis. This study retrieved records for hospitalized children aged under 18 years and diagnosed with ITP from the hospital registry in a tertiary university hospital. Clinical variables were recorded by reviewing the medical records with structured data entry for ITP admission. The serial follow-up platelet counts within 12 months after diagnosis were recorded. The times of ITP remission were identified by experienced pediatric hematologists. Patients with mild-form ITP (platelet counts ≥ 30 × 10^9^/L) at diagnosis or who were lost to follow-up within 3 months were excluded. From 1988 to 2019, 546 children were enrolled, and a total of 497 children with severe ITP were included in the further analysis. In total, one (0.2%) died of an intracranial hemorrhage, 363 (73.2%) children went into remission at 3 months, 40 (8.1%) went into remission between 6 and 12 months, and 104 (20.9%) developed chronic ITP. The shared significant predictors for remission by the third, sixth, and twelfth months included pre-adolescent age (<10 years) at diagnosis, abrupt onset (duration of symptoms prior to admission ≤ 2 weeks), and speedy recovery (platelet count > 100 × 10^9^/L at 1 month post diagnosis). ITP patients with positive viral serology tests or vaccination within 4 weeks had trends of delayed remission. In conclusion, diagnosis before preadolescent age, abrupt onset, and speedy recovery may share favorable factors for the remission of childhood ITP assessed at different timepoints.

## 1. Introduction

Immune thrombocytopenia (ITP, platelet counts < 100 × 10^9^/L) is the most common acquired childhood bleeding disorder, clinically characterized by a low platelet count in the absence of other thrombocytopenia causes [1,2]. Most children present with a typical history of acute purpura and bruising after a mild viral infection [3,4,5]. ITP in children is usually a benign course with speedy recovery [6]. However, 3–5% of children with ITP face a greater risk of bleeding, resulting in significant morbidity and mortality [7], and children with severe thrombocytopenia (platelet counts below the threshold of approximately 30 × 10^9^/L) require investigation and treatment. Importantly, about 20% of children with newly diagnosed ITP may develop chronic thrombocytopenia status (e.g., platelet counts < 100 × 10^9^/L persisting after 12 months) [3,8,9,10,11].

Families of children with severe ITP often live with fear and anxiety since their children have a greater likelihood of needing rehospitalization and treatment [12,13]. Early identifying predictors associated with the resolution of ITP at the time of diagnosis may be helpful for family guidance to ease anxiety and improve the quality of life of the family.

Several studies have attempted to identify clinical and laboratory features to predict disease resolution in pediatric ITP [3,9,10,11,14,15,16,17,18,19,20,21,22,23]. A study conducted in Nordic countries found six parameters associated with remission at 3 and 6 months, including age, sex, onset type, preceding infection, bleeding severity, and platelet count at diagnosis [19,24]. The Intercontinental Cooperative ITP Study Group Registry II found that age, bleeding severity, and initial treatment with a combination of corticosteroids and IVIG were associated with remission at 12 months [16]. A systemic review and meta-analysis identified predictors including age, sex, onset type, preceding infection or vaccination, platelet counts at diagnosis, and treatment with IVIG alone [18]. However, most research has considered all forms of ITP [18], including children with less severe thrombocytopenia (e.g., platelet counts 30–100 × 10^9^/L), and limited research has focused explicitly on ITP in children with severe thrombocytopenia. Due to the variations in the definitions of remission and chronic ITP among studies, the identification of predictors for different timepoints of remission deserves more research attention for children with severe ITP with platelet counts of <30 × 10^9^/L.

The aim of the study was to analyze the course and clinical outcomes of children with severe ITP who received diagnosis, treatment, and one-year follow-up at a single university hospital. This study also attempted to correlate clinical factors at diagnosis with disease remission (platelet counts ≥ 100 × 10^9^/L) at three arbitrarily set timepoints of 3, 6, and 12 months after diagnosis.

## 2. Materials and Methods

### 2.1. Study Setting and Design

The study unit was a 40-bed tertiary pediatric hematologic ward at the National Cheng Kung University Hospital in Tainan, Taiwan. Ethical approval according to the guidelines of the Declaration of Helsinki was obtained by the Institutional Review Board of National Cheng Kung University Hospital (A-ER-111-141, 7 June 2022).

We retrospectively reviewed the medical records of those diagnosed with ITP in the hospitalization registry of the Department of Pediatrics between 1988 and 2019.

### 2.2. Institute Treatment Protocol of ITP

Travel history, family history, clinical history, serology, and vaccination were reviewed with a structured form at admission. The treatment of ITP was based on institutional protocols and physician preference. If a bone marrow examination showed no malignancy or megakaryocytic hypoplasia and patients did not have a severe infection, methylprednisolone was administered in doses of 30 mg/kg (maximum 1 g) for three consecutive days [25,26,27].

If patients had minor bleeding symptoms or had not been hospitalized, they did not routinely require treatment or were given oral prednisolone 2 mg/kg/day at the outpatient clinic, tapering off in 3–4 weeks [28]. If patients had severe bleeding or if bone marrow pathology had not yet been excluded, treatment with intravenous immunoglobulin (IVIG) 0.4 mg/kg/day for five consecutive days or 1 g/kg for two days was administered [25,29,30].

After initial management, patients were scheduled for a follow-up appointment. The institutional follow-up protocols suggest follow-up platelet counts weekly for 1 month after diagnosis, then monthly for 1–6 months, and finally every 3 months for 7–12 months after diagnosis. However, if patients’ platelet counts do not recover, they are continuously followed up according to the judgment of the physician, even up to 10 years after diagnosis.

### 2.3. Inclusion and Exclusion Criteria

#### 2.3.1. Inclusion Criteria

We included hospitalized children with a diagnosis of ITP and aged under 18 years. We only included children with a platelet count at diagnosis of <30 × 10^9^/L, namely, severe ITP. Platelet counts were determined using an automated cell counter (Coulter Electronics), and the samples with thrombocytopenia were confirmed routinely by medical technicians with microscopic examination of the blood smears to exclude pseudo-thrombocytopenia caused by artefact clumps, platelet clumps in the EDTA sample, etc.

#### 2.3.2. Exclusion Criteria

Patients were excluded if they were lost to follow-up within 3 months after diagnosis without a record of remission. Patients who developed hematological malignancy during follow-up were also excluded.

### 2.4. Clinical Variables and Covariate Definitions

The data relating to the initial presentation were recorded for each patient, including age, sex, history of recent infection or vaccination, the onset of symptoms, the severity of bleeding symptoms, platelet count at diagnosis, surveillance of viral etiology (i.e., cytomegalovirus, Epstein–Barr virus, rubella, hepatitis B, hepatitis C, and herpes simplex virus), and immunological markers, i.e., antinuclear antibody (ANA), C3, and C4. We also recorded the type of initial treatment, the response, and the follow-up platelet counts until remission. The children’s ages were categorized into three groups: <18 months, 18 months–10 years, and >10 years.

A history of recent infection or vaccination was defined as a recorded infection of the upper respiratory or gastrointestinal tract or vaccination within 4 weeks before diagnosing ITP.

The onset of symptoms was categorized as abrupt onset (<15 days) or insidious onset (≥15 days).

The severity of bleeding symptoms was categorized into two groups according to the international consensus report: minor/mild and moderate/severe [31]. The platelet count at diagnosis was categorized into three groups: <5 × 10^9^/L, 5–20 × 10^9^/L, and 20–29 × 10^9^/L.

### 2.5. Outcomes

The primary outcome was the remission of ITP at the three arbitrarily set timepoints of 3, 6, and 12 months. Remission was defined as a platelet count of ≥100 × 10^9^/L without treatment for at least 2 months. We recorded the first timepoint of achieving remission, and the time of completed remission was confirmed by experienced pediatric hematologists. This study arbitrarily defined early remission as remission at 3 months after diagnosis and delayed remission as that between 7 and 12 months after diagnosis.

### 2.6. Statistical Analysis

The statistical analysis was carried out using MedCalc Statistical Software version 18.2.1. Missing data were not included in the analysis. Categorical variables, i.e., age (categorized as <18 months, 18 months–10 years, and >10 years), sex, platelet count at diagnosis (categorized as <5 × 10^9^/L, 5–20 × 10^9^/L, and 20–29 × 10^9^/L), preceding infection (yes and no), bleeding severity (minor/mild and moderate/severe), onset type (abrupt and insidious), positive viral serology test or vaccination (yes and no), first-line treatment (observation, oral prednisolone, pulse methylprednisolone, and IVIG), and second-line therapy (yes and no), were analyzed by the chi-square test.

Variables with *p*-values of <0.2 from the univariate analysis were included in the multivariate logistic regression model to assess independent predictors of the remission of ITP. A cut-off of *p* < 0.2 is commonly used for retaining significant variables in univariate analysis in the building of a multivariate logistic regression model [14,15,16]. A stepwise elimination method based on the likelihood ratio test was used to select the final model. The odds ratio (OR) and 95% confidence interval (CI) were used to determine the increased relative risk, and *p*-values of <0.05 were considered statistically significant.

## 3. Results

### 3.1. Demographic Characteristics

**Figure 1:**
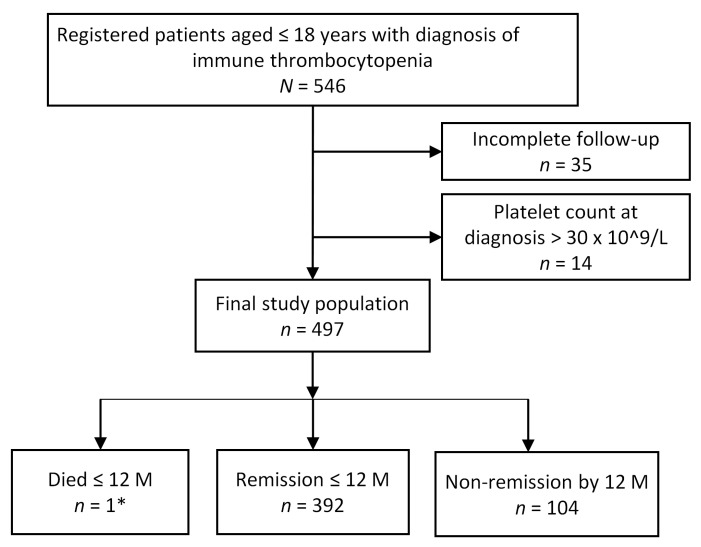
Patient enrollment, follow-up, and outcome. * Intracranial hemorrhage.

A total of 546 *hospitalized* children diagnosed with ITP at our hospital were identified. Forty-nine children were excluded from the analysis because of a platelet count at diagnosis of more than 30 × 10^9^ or incomplete follow-up data (Figure 1).

Of the 497 children, 352 (71.0%) children achieved remission by 6 months, 40 (8.1%) children had chronic ITP according to the old definition and achieved remission at 7–12 months from diagnosis, and 104 (20.9%) had thrombocytopenia at 12 months post diagnosis (chronic ITP). One patient (0.2%) died of an intracranial hemorrhage following a head injury 6 months after diagnosis. The demographic and clinical characteristics of the study group are presented in Table 1.

The most common findings at the physical examination were petechial/ecchymosis and oral mucosa bleeding. Severe bleeding leading to a decrease in hemoglobin of >2 g/dL or internal bleeding was noted in 36 patients (7.2%), and intracranial hemorrhage was observed in 5 patients (1%) (six events).

A history of viral infection or vaccination within 4 weeks before diagnosis was recorded in 303 patients (61.0%). In total, 32 patients (6.4%) had a recent vaccination history, and 141 (28.4%) had a positive serological viral infection. Among the 141 patients with a positive serological viral infection, the top three were cytomegalovirus (CMV) in 54 patients (10.9%), Epstein–Barr virus (EBV) in 46 patients (9.3%), and rubella in 16 patients (3.2%). Autoimmune profiles were not performed routinely and seldom checked among patients younger than three years old. ANA, C3, and C4 were checked in only 270 patients (54.3%) and were not included in the analysis. The treatment of ITP was based on institutional protocols and physician preference. In total, 479 patients (96.4%) received treatment, 155 patients (31.2%) received IVIG 1 g/kg for 2 days, 250 patients (50.3%) received high-dose methylprednisolone pulse therapy 30 mg/kg (maximum 1 g) for 3 days, and 74 patients (14.9%) received oral prednisolone 2 mg/kg/day, tapered off in 3–4 weeks. Children who were initially observed without therapy had higher platelet counts and minor bleeding symptoms. The physicians preferred IVIG therapy for younger children with severe bleeding symptoms. Of the 479 who received treatment, 184 patients (37.0%) had no response to the initial treatment or failed to maintain a platelet count and received second-line treatment or more. Of the patients, 352 (70.8%) had a platelet count of more than 100 × 10^9^/L at one month, and 363 (73.0%) had a platelet count of more than 100 × 10^9^/L at 3 months post diagnosis.

### 3.2. Predictive Factors for Remission at 12 Months (Acute ITP)

The univariate analysis of the demographic and clinical characteristics between the 392 children in remission and the 104 children not in remission is presented in Table 2.

The children’s ages were initially categorized into three groups, and they were finally categorized into two groups, <10 years and ≥10 years, because the remission rates in the groups <18 months and 18 months–10 years were similar. The platelet counts at diagnosis were also categorized into two groups, <20 × 10^9^/L and ≥20 × 10^9^/L, for the same reason. The significant predictors for acute ITP were an age < 10 years (*p* < 0.001), a platelet count at diagnosis of <20 × 10^9^/L (*p* = 0.003), male gender (*p* = 0.011), a history of recent infection (*p* < 0.001), abrupt onset of symptoms (*p* < 0.001), and positive serological viral infection or vaccination (*p* < 0.001). The severity of bleeding had no statistically significant association with remission (*p* = 0.156). In the univariate analysis, children who were initially treated with IVIG had a higher rate of remission (*p* = 0.006). The platelet counts > 100 × 10^9^/L at 1 month post diagnosis had a statistically significant association with acute ITP (*p* < 0.001).

### 3.3. Predictors for Disease Resolution at Different Timepoints

Table 3 shows the univariate analysis of the predictors of remission at 3, 6, and 12 months. The most significant predictors for the remission of ITP at these three timepoints were an age < 10 years (*p* < 0.001) and the onset of bleeding symptoms in <15 days (*p* < 0.001). A history of recent infection, positive serological viral infection or vaccination, and first-line IVIG therapy were also statistically associated with remission at these three timepoints.

### 3.4. Multivariate Analysis for Determining Independent Predictors

The multivariate analysis with logistic regression results for determining the independent predictors of disease remission is presented in Table 4. The predictive factors of remission at 12 months were age, onset type, and positive serological viral infection or vaccination. Children aged < 10 years had a higher likelihood (OR: 3.35, 95% CI: 1.76–6.38) of disease remission compared to those aged ≥ 10 years. Children with abrupt onset had a higher likelihood of disease remission compared to those with insidious onset (OR: 9.83, 95% CI: 5.67–17.05). Children who had positive serological viral infection or vaccination had a higher likelihood of disease remission assessed at 12 months (OR: 2.21, 95% CI: 1.14–4.28). However, a lower platelet count at diagnosis, gender, history of recent infection, and IVIG therapy were not associated with disease remission at 12 months.

At 6 months post diagnosis, the independent predictive factors for remission were an age < 10 years (OR: 3.89, 95% CI: 2.07–7.34), male sex (OR: 1.72, 95% CI: 1.07–2.75), abrupt onset (OR: 7.58, 95% CI: 4.48–12.83), and a platelet count < 5 × 10^9^/L at diagnosis (OR: 1.72, 95% CI: 1.02–2.88). At 3 months post diagnosis, children aged < 10 years (OR: 2.27, 95% CI: 1.26–4.08) and those with abrupt onset (OR: 5.46, 95% CI: 3.32–8.96) had the highest remission rates. These two factors were also the most significant predictors of the resolution of ITP at all timepoints.

Among the 392 acute ITP patients, a multivariate analysis comparing the predictive factors between the 352 children who went into remission within 6 months and the 40 who went into remission at 7–12 months showed that a younger age, a lower platelet count at diagnosis, and abrupt onset had a higher likelihood of disease resolution within 6 months.

## 4. Discussion

ITP in children is typically a self-limited disorder that resolves within several weeks to months. About 80% of newly diagnosed ITP children undergo spontaneous recovery, and only 20% have persistent thrombocytopenia for >12 months after diagnosis, which is then classified as chronic ITP. However, the estimated incidence of intracranial hemorrhage is 0.19–0.78% [32]. Therefore, families of children with ITP often live with fear and anxiety. Many studies have found various clinical factors that can predict whether a patient will recover within 6 or 12 months or whether it will become a chronic condition [14,15,16,18,24,33]. These studies are summarized in Supplementary Table S1. Providing information on such predictive factors may be beneficial for patients and parents and for physicians who provide counseling to reduce the anxiety of patients and parents. However, these studies included children with less severe thrombocytopenia, except for the NOPHO register cohort. Our study provides prediction information for children with severe thrombocytopenia (with platelet counts < 30 × 10^9^/L).

A total of 392 (78.9%) of our patients achieved remission at 12 months from diagnosis. Using history and clinical features at diagnosis and platelet counts at 1 month post diagnosis, we found that the remission of ITP at 12 months was associated with an abrupt onset of symptoms (<15 days), younger age (<10 years), and positivity for viral serological tests and a vaccination history. These findings are compatible with most of the literature [10,11,14,15,18,19,21]. Our study also showed that patients with positive viral serology tests or vaccination had trends of achieving disease remission between 7 and 12 months post diagnosis. However, our study also showed that the severity of bleeding had no statistically significant association with remission, and IVIG therapy was not an independent predictive factor for remission in the multivariate analysis of the findings in the Intercontinental Cooperative ITP Study Group Registry [16,34].

Platelet counts of more than 100 × 10^9^/L at 1 month post diagnosis had a higher likelihood of disease remission. Although it is possible to interpret this as a treatment effect, we believe that this was due to the natural course of the disease. As we know, the response duration of IVIG or pulse methylprednisolone therapy for chronic ITP lasts less than 1 month, and chronic ITP patients must receive treatment continuously to maintain platelet counts in acceptable ranges. Thus, we attribute platelet counts of ≥100 × 10^9^/L at 1 month after initial treatment to the natural course of the disease and not to the initial therapy effect.

The effect of initial therapy on the rate of resolution is debated. Some studies have suggested that treatment with IVIG might result in a higher rate of remission [16,34]. However, the differences in therapy response in different age groups should be considered. Infants were more likely to be treated with IVIG, whereas corticosteroids were used most frequently in older children and adolescents [3]. In our study, there were 155 (31.2%) children who received IVIG as the initial therapy. Most were younger than 18 months with lower platelet counts at diagnosis. The remission of ITP at 3, 6, and 12 months, and beyond 12 months from diagnosis, adjusted for age, gender, onset type, and platelet count at diagnosis, was not different between children treated with first-line IVIG compared to those treated otherwise.

Predictive clinical factors for disease remission have varied among studies. Factors that can be easily identified with high specificity are essential for use in a predictive model. However, these factors differ among studies. Recently, Schmidt et al. published a clinical prediction score (a calculator is available online at http://www.itprecoveryscore.org, accessed on 1 December 2022) to differentiate transient vs. persistent disease courses in newly diagnosed childhood ITP. They used clinical variables including age, platelet count, gender, history of infection, vaccination, bleeding symptoms, and disease onset. It was developed with the Nordic Pediatric Hematology–Oncology (NOPHO) cohort in Nordic countries and validated with the Treatment with or without Intravenous Immunoglobulins in Kids with ITP (TIKI) cohort in the Netherlands [24]. Our study is the first such study conducted in Taiwan, and we found that the above variables were significant predictive factors for disease resolution except for bleeding symptoms. Our findings support that the clinical prediction score applies to ITP children in our country for medical decisions.

### Strengths and Limitations

The strengths of this study were the cohort’s relatively large number of ITP children (*n* = 532) and the high overall follow-up rate of 93.2% at 12 months. Our study was limited by its retrospective nature. The study also covered a 31-year duration from 1988 to 2019. The definition, treatment, and management of ITP changed during this period. The timing of patient follow-up and treatment options also varied.

## 5. Conclusions

In conclusion, our study showed that abrupt onset, age < 10 years, and a follow-up platelet count of more than 100 × 10^9^/L at 1 month are significant and independent factors for predicting disease remission at 3, 6, and 12 months. Patients with positive viral serology tests or vaccination had trends of delayed remission. Providing this information should help reduce anxiety and improve the quality of life for patients and their families with newly diagnosed immune thrombocytopenia.

## Figures and Tables

**Table 1:** Demographic and clinical characteristics of children with ITP.

Total *N*	497
Age at diagnosis, median (IQR) [range] (years)	3.8 (1.4–7.6) [0.1–17.9]
<1.5, *n* (%)	130 (26.2%)
≥1.5–10, *n* (%)	286 (57.5%)
≥10, *n* (%)	81 (16.3%)
Platelet count at diagnosis, median (IQR) [range] (×10^9^/L)	6 (3–13) [1,2,3,4,5,6,7,8,9,10,11,12,13,14,15,16,17,18,19,20,21,22,23,24,25,26,27,28,29]
<5 × 10^9^/L	183 (36.8%)
≥5–20 × 10^9^/L	262 (52.7%)
≥20–29 × 10^9^/L	52 (10.5%)
Sex	
Male, *n* (%)	266 (53.5%)
Female, *n* (%)	231 (46.5%)
History of recent infection	
Yes, *n* (%)	303 (61.0%)
No, *n* (%)	184 (37.0%)
Missing data, *n* (%)	10 (2.0%)
Onset type	
Abrupt onset, *n* (%)	372 (74.8%)
Insidious onset, *n* (%)	98 (19.7%)
Missing data, *n* (%)	27 (5.4%)
Bleeding severity at diagnosis	
Minor/mild, *n* (%)	221 (44.5%)
Moderate/severe, *n* (%)	267 (53.7%)
Missing data, *n* (%)	9 (1.8%)
Positive viral serology/vaccination	173 (34.8%)
Vaccination within 4 weeks, *n* (%)	32 (6.4%)
CMV-IgM (+) or urine antigen (+), *n* (%)	54 (10.9%)
EBV-IgM (+) or EBV-IgG ≥ 1:1280, *n* (%)	46 (9.3%)
Rubella-IgM (+), *n* (%)	16 (3.2%)
Other viruses, *n* (%)	25 (5.0%)
ANA at diagnosis	
Negative, *n* (%)	220 (44.3%)
Positive ≥ 1:80 (+), *n* (%)	50 (10.1%)
Not determined, *n* (%)	227 (45.7%)
Treatment at diagnosis	
Observation, *n* (%)	18 (3.6%)
Oral prednisolone, *n* (%)	74 (14.9%)
Pulse methylprednisolone, *n* (%)	250 (50.3%)
Intravenous immunoglobulin, *n* (%)	155 (31.2%)
Second-line therapy	
Yes	184 (37.0%)
No	313 (63.0%)
Platelet count at 1 month (×10^9^/L)	
≥100 × 10^9^/L	352 (70.8%)
<100 × 10^9^/L	135 (27.2%)
Missing data	10 (2%)
Platelet count at 3 months (×10^9^/L)	
≥100 × 10^9^/L	363 (73.0%)
<100 × 10^9^/L	134 (27.0%)
Platelet count at 6 months (×10^9^/L)	
≥100 × 10^9^/L	352 (70.8%)
<100 × 10^9^/L	145 (29.2%)
Platelet count at 12 months (×10^9^/L)	
≥100 × 10^9^/L	392 (78.9%)
<100 × 10^9^/L	104 (20.9%)
Death due to intracranial hemorrhage	1 (0.2%)

IQR, interquartile range; CMV, cytomegalovirus; EBV, Epstein–Barr virus; IgM, immunoglobulin M; IgG, immunoglobulin G; ANA, antinuclear antibody.

**Table 2:** Univariate predictors of remission by 12 months for children with ITP.

	Remission by 12 Months Post Diagnosis		*p*-Value
	Yes, *n* = 392 (78.9%)	No, *n* = 104 (20.9%)	
Age at diagnosis, median (IQR) (years)	3.1 (1.2–6.1)	7.1 (3.5–11.9)	<0.001
<10, *n* (%)	121 (93.1%)	9 (6.9%)	
1.5–10, *n* (%)	232 (81.1%)	54 (18.9%)	
≥10, *n* (%)	39 (48.7%)	41 (51.2%)	
Platelet count at diagnosis, median (IQR)	6 (3–12)	8 (4–15)	0.003
<5 × 10^9^/L, *n* (%)	152 (83.5%)	30 (16.5%)	
5–20 × 10^9^/L, *n* (%)	208 (79.4%)	54 (20.6%)	
20–29 × 10^9^/L, *n* (%)	32 (61.5%)	20 (38.5%)	
Sex			0.011
Male, *n* (%)	221 (83.4%)	44 (16.6%)	
Female, *n* (%)	171 (74.0%)	60 (26.0%)	
History of recent infection			<0.001
Yes, *n* (%)	259 (85.5%)	44 (14.5%)	
No, *n* (%)	129 (70.1%)	55 (29.9%)	
Missing data, *n* (%)	4 (44.4%)	5 (55.6%)	
Onset type			<0.001
Abrupt onset, *n* (%)	336 (90.3%)	36 (9.7%)	
Insidious onset, *n* (%)	41 (42.3%)	56 (57.7%)	
Missing data, *n* (%)	15 (55.6%)	12 (44.4%)	
Bleeding severity at diagnosis			0.156
Moderate/severe, *n* (%)	219 (82.0%)	48 (18.0%)	
Minor/mild, *n* (%)	169 (76.8%)	51 (23.2%)	
Missing data, *n* (%)	4 (44.4%)	5 (55.6%)	
Positive viral serology/vaccination			<0.001
Yes, *n* (%)	154 (89.0%)	19 (11.0%)	
No, *n* (%)	238 (73.7%)	85 (26.3%)	
Treatment at diagnosis			0.006
IVIG, *n* (%)	134 (86.5%)	21 (13.5%)	
Others, *n* (%)	258 (75.7%)	83 (24.3%)	
Needed second-line therapy			<0.001
No, *n* (%)	269 (85.9%)	44 (14.1%)	
Yes, *n* (%)	123 (67.2%)	60 (32.8%)	
Platelet count at 1 month			<0.001
≥100 × 10^9^/L, *n* (%)	321 (91.2%)	31 (8.8%)	
<100 × 10^9^/L, *n* (%)	68 (50.7%)	66 (49.3%)	
Missing data, *n* (%)	3 (30%)	7 (70%)	
Platelet count at 3 months			<0.001
≥100 × 10^9^/L, *n* (%)	346 (95.3%)	17 (4.7%)	
<100 × 10^9^/L, *n* (%)	46 (34.6%)	87 (65.4%)	

IQR, interquartile range; IVIG, intravenous immunoglobulin; *p* according to chi-square test.

**Table 3:** Rates and predictors for remission in childhood ITP according to time from diagnosis.

Univariate Analysis	Remission by 3 Months 363/496 (73.2%)		Remission by 6 Months 352/496 (71.0%)		Remission by 12 Months 392/496 (79.0%)	
	OR (95% CI)	*p*-Value	OR (95% CI)	*p*-Value	OR (95% CI)	*p*-Value
Age < 10 years	3.26 (1.99–5.35)	<0.001	5.00 (1.46–2.53)	<0.001	5.89 (3.52–9.85)	<0.001
Platelet count at diagnosis < 5 × 10^9^/L	1.49 (0.97–2.29)	0.065	1.93 (1.26–2.96)	0.003	1.56 (0.98–2.52)	0.063
Platelet count at diagnosis < 20 × 10^9^/L	1.83 (1.01–3.33)	0.048	1.77 (0.98–3.19)	0.059	2.68 (1.46–4.92)	0.002
Male	1.45 (0.98–2.16)	0.066	1.88 (1.27–2.78)	0.002	1.76 (1.14–2.73)	0.011
History of recent infection	1.79 (1.19–2.69)	0.005	2.12 (1.42–3.17)	<0.001	2.51 (1.60–3.93)	<0.001
Abrupt onset	6.33 (3.91–10.26)	<0.001	9.78 (5.92–16.14)	<0.001	12.75 (7.51–21.65)	<0.001
Bleeding severity at diagnosis: moderate/severe	1.45 (0.97–2.17)	0.073	1.54 (1.04–2.29)	0.032	1.38 (0.88–2.14)	0.156
Positive viral serology/vaccination	1.72 (1.11–2.67)	0.016	1.91 (1.24–2.95)	0.003	2.89 (1.69–4.95)	<0.001
First-line IVIG therapy	1.62 (1.03–2.54)	0.038	1.69 (1.09–2.64)	0.020	2.05 (1.22–3.46)	0.006
Needed second-line therapy	0.25 (0.17–0.38)	<0.001	0.29 (0.19–0.44)	<0.001	0.34 (0.22–0.52)	<0.001
Platelet count at 1 month ≥100 × 10^9^/L	15.76 (9.64–25.77)	<0.001	12.53 (7.83–20.05)	<0.001	10.05 (6.09–16.58)	<0.001

OR, odds ratio; IVIG, intravenous immunoglobulin.

**Table 4:** Multivariate logistic regression analysis for predictors of remission in childhood ITP according to time from diagnosis.

*n*/Total *N* (%)	Remission by 3 Months 363/496 (73.2%)		Remission by 6 Months 352/496 (71.0%)		Remission by 12 Months 392/496 (79.0%)	
	OR (95% CI)	*p*-Value	OR (95% CI)	*p*-Value	OR (95% CI)	*p*-Value
Age < 10 years	2.27 (1.26–4.08)	0.006	3.89 (2.07–7.34)	<0.001	3.35 (1.76–6.38)	<0.001
Platelet count at diagnosis < 5 × 10^9^/L	-	-	1.72 (1.02–2.88)	0.041	-	-
Platelet count at diagnosis < 20 × 10^9^/L	-	-	-	-	-	-
Male	-	-	1.72 (1.07–2.75)	0.025	-	-
History of recent infection	-	-	-	-	-	-
Abrupt onset	5.46 (3.32–8.96)	<0.001	7.58 (4.48–12.83)	<0.001	9.83 (5.67–17.05)	<0.001
Bleeding severity at diagnosis: moderate/severe	-	-	-	-	-	-
Positive viral serology/vaccination	-	-	-	-	2.21 (1.14–4.28)	0.018
First-line IVIG therapy	-	-	-	-	-	-

OR, odds ratio; IVIG, intravenous immunoglobulin, -: variables enrolled in statistical model but nonsignificant.

## Data Availability

The corresponding author had full access to the dataset used and analyzed during the current study. The datasets used during the current study are available from the corresponding author upon reasonable request.

## Supplementary Materials

The following supporting information can be downloaded at: https://www.mdpi.com/article/10.3390/diagnostics13030341/s1, Table S1: Identified clinical and laboratory predicting markers of pediatric ITP (review of literature).
