# Supplementary material for: Predictors of Remission in Severe Childhood Immune Thrombocytopenia
Source: Diagnostics (Basel). 2023 Jan 17;13(3):341. doi: 10.3390/diagnostics13030341 (PMC9914323; doi:10.3390/diagnostics13030341)
Supplement: Supplementary file 1 [file diagnostics-13-00341-s001.zip › diagnostics-2111588-supplementary.pdf]

Supplementary Table S1. Identified clinical and laboratory predicting markers of pediatric ITP (review of literature)

Ref and year	Population	Number (evaluated)	Time point	Predictors	Study design and Statistical methods
#3<br>2003	ICIS registry<br>Worldwide<br><br>Platelet count < 150 x 10^9/L	2540<br>(1742)	6 months	Age	Prospective registry<br><br>Multivariate Logistic regression
#9<br>2004	Single center<br>Unite States<br><br>Platelet count < 70 x 10^9/L	103	6 months	Mean platelet volume<br>Preceding infection	Retrospective<br><br>Multivariate Logistic regression
#10<br>2005	NOPHO registry<br>Nordic country<br><br>Platelet count < 30 x 10^9/L	506<br>(423)	6 months	Onset type	Prospective registry<br>Multivariate Logistic regression
#19<br>2007	NOPHO registry<br>The same with ref #10	506<br>(233 in training)<br>(144 in validate cohort)	6 months	Nordic scoring:<br><br>Age<br>Sex<br>Preceding infection<br>Onset type<br>Platelet at diagnosis<br>Bleeding severity	Prospective registry<br><br>Univariate<br>The Odds ratio in univariate converted to predictive weights
#24<br>2021	NOPHO registry<br>The same with ref #10<br><br>TIKI registry<br>Similar to the NOPHO	506<br>(377 in training)<br><br>206<br>(194 in external	3 months	Prediction score:<br><br>Age<br>Sex<br>Preceding infection	Prospective registry<br><br>Binomial Logistic regression fitted with ridge regression using glmnet

	registry	validation)		Preceding vaccines<br>Onset type<br>Platelet at diagnosis<br>Bleeding severity	
#11<br>2008	Multi-center<br>Unite States<br><br>Platelet count < 50 x 10^9/L	259	6 months	Age<br>Platelet at diagnosis<br>Onset type<br>Bleeding severity	Retrospective<br><br>Parametric survival regression
#20<br>2009	Multi-center<br>Argentina<br><br>Platelet count < 150 x 10^9/L	1683<br>(1418)	6 months	Age<br>Preceding infection<br>Platelet at diagnosis<br>Nordic score	Retrospective<br><br>Univariate
#21<br>2010	Single center<br>Egypt<br><br>Platelet count < 150 x 10^9/L	409<br>(344)	6 months	Age<br>Onset type<br>Platelet at diagnosis	Retrospective<br><br>Univariate
#23<br>2010	Multi-center<br>Japan<br><br>Platelet count < 150 x 10^9/L	247	6 months	Age<br>Preceding infection<br>Platelet at diagnosis<br>Treatment	Retrospective<br><br>Multivariate Logistic regression
#15<br>2017	Single center<br>Thailand<br><br>Platelet count < 100 x 10^9/L	417<br>(405)	12 months	Age<br>Onset type<br>Platelet at 4-week postdiagnosis	Retrospective<br><br>Multivariate Logistic regression

#14<br>2013	Single center<br>Israel<br><br>Platelet count < 100 x 10^9/L	472<br>(309)	3 months	Age<br>Onset type<br>Platelet at diagnosis	Retrospective<br><br>Multivariate Logistic regression
		472<br>(465)	6 months	Age<br>Onset type<br>Platelet at diagnosis	
		472<br>(312)	12 months	Age<br>Onset type<br>Jewish origin	
#16<br>2018	ICIS-II registry<br>Worldwide<br><br>Platelet count < 128 x 10^9/L	1345<br>(705)	12 months	Age<br>Bleeding severity<br>Treatment	Prospective registry<br><br>Multivariate Logistic regression
		1345<br>(383)	24 months	Age<br>Treatment	
#17<br>2019	Single center<br>Turkey<br><br>Platelet count < 100 x 10^9/L	211	12 months	Age<br>Sex<br>Preceding infection<br>Platelet at diagnosis	Retrospective<br><br>Univariate Chi-Square analysis
#18<br>2014	Meta-analysis<br>Worldwide<br><br>Platelet count < 150 x 10^9/L	54 studies (43–1984)	6 months (12 months in later articles)	Age<br>Sex<br>Preceding infection<br>Onset type<br>Platelet at diagnosis<br>Positive ANA<br>Treatment<br>Bleeding severity	Pooled meta-analysis
